# Supplementary figures and images for: Hepatitis B virus hijacks MRE11–RAD50–NBS1 complex to form its minichromosome
Source: PLoS Pathog. 2025 Jan 3;21(1):e1012824. doi: 10.1371/journal.ppat.1012824 (PMC11734937; doi:10.1371/journal.ppat.1012824)

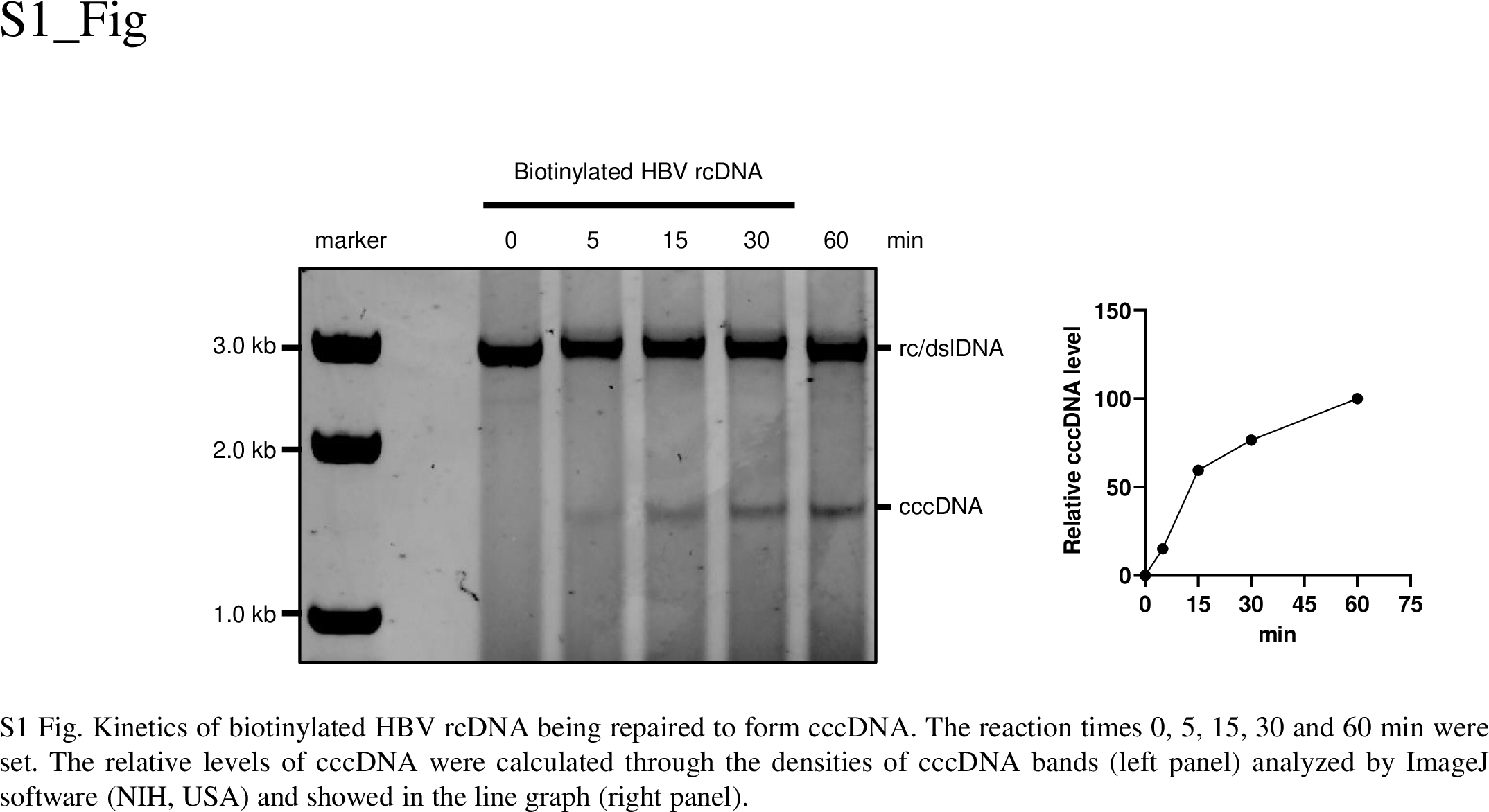

Supplement: S1 Fig — The reaction times 0, 5, 15, 30 and 60 min were set. The relative levels of cccDNA were calculated through the densities of cccDNA bands (left panel) analyzed by ImageJ software (NIH, USA) and showed in the line graph (right panel). (TIF) [file ppat.1012824.s001.tif]

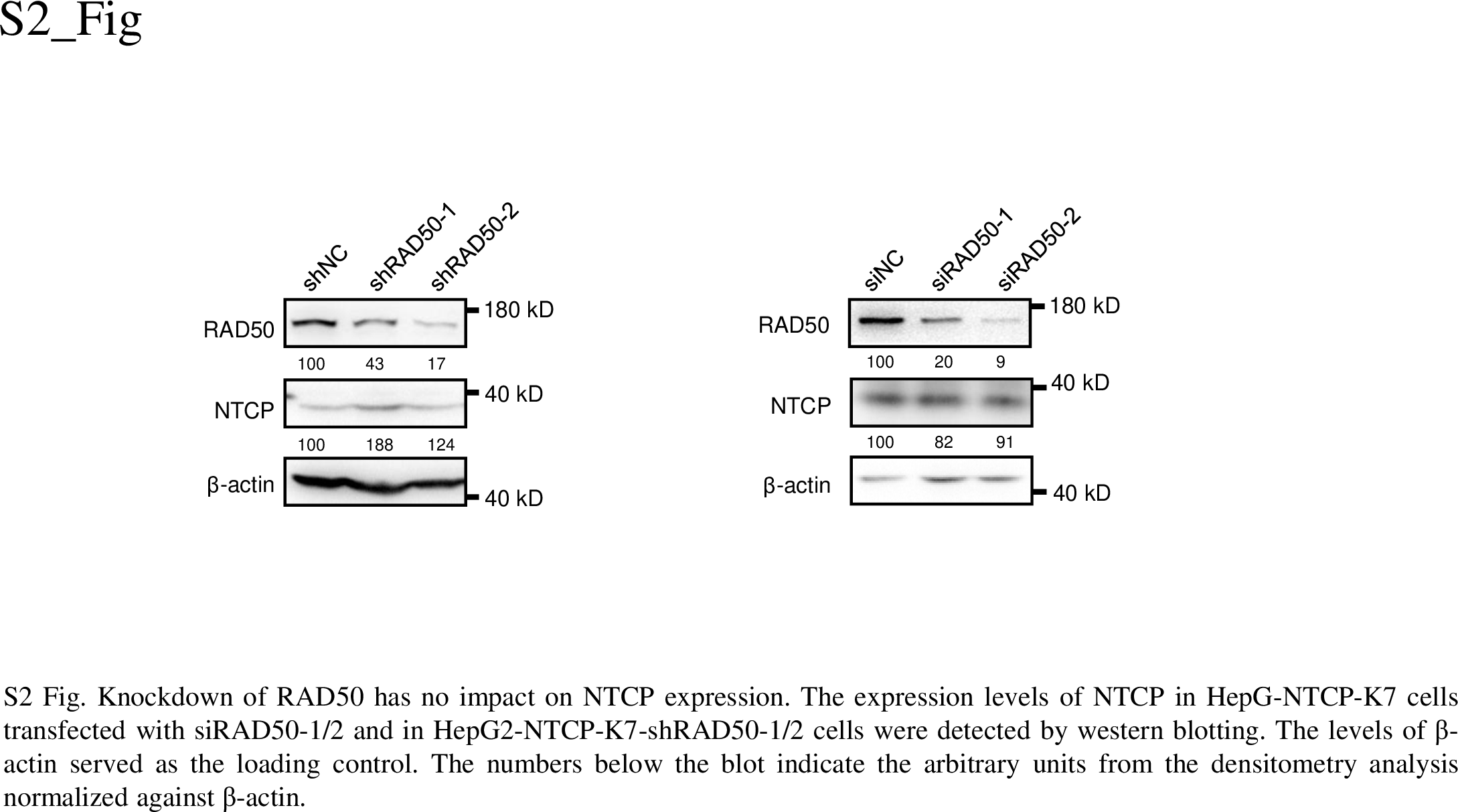

Supplement: S2 Fig — The expression levels of NTCP in HepG-NTCP-K7 cells transfected with siRAD50-1/2 and in HepG2-NTCP-K7-shRAD50-1/2 cells were detected by western blotting. The levels of β-actin served as the loading control. The numbers below the blot indicate the arbitrary units from the densitometry analysis normalized against β-actin. (TIF) [file ppat.1012824.s002.tif]

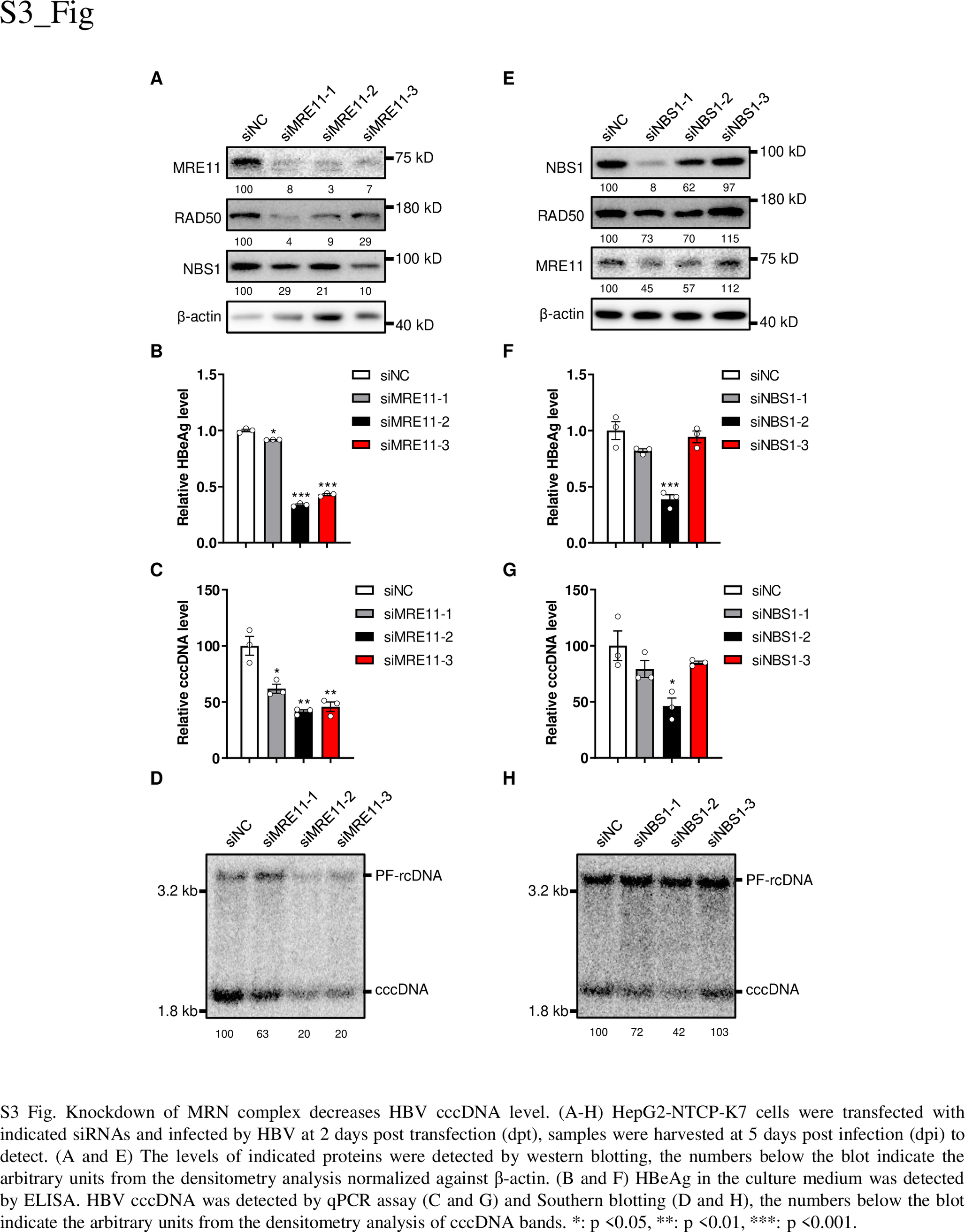

Supplement: S3 Fig — (A-H) HepG2-NTCP-K7 cells were transfected with indicated siRNAs and infected by HBV at 2 days post transfection (dpt), samples were harvested at 5 days post infection (dpi) to detect. (A and E) The levels of indicated proteins were detected by western blotting, the numbers below the blot indicate the arbitrary units from the densitometry analysis normalized against β-actin. (B and F) HBeAg in the culture medium was detected by ELISA. HBV cccDNA was detected by qPCR assay (C and G) and Southern blotting (D and H), the numbers below the blot indicate the arbitrary units from the densitometry analysis of cccDNA bands. *: p <0.05, **: p <0.01, ***: p <0.001. (TIF) [file ppat.1012824.s003.tif]

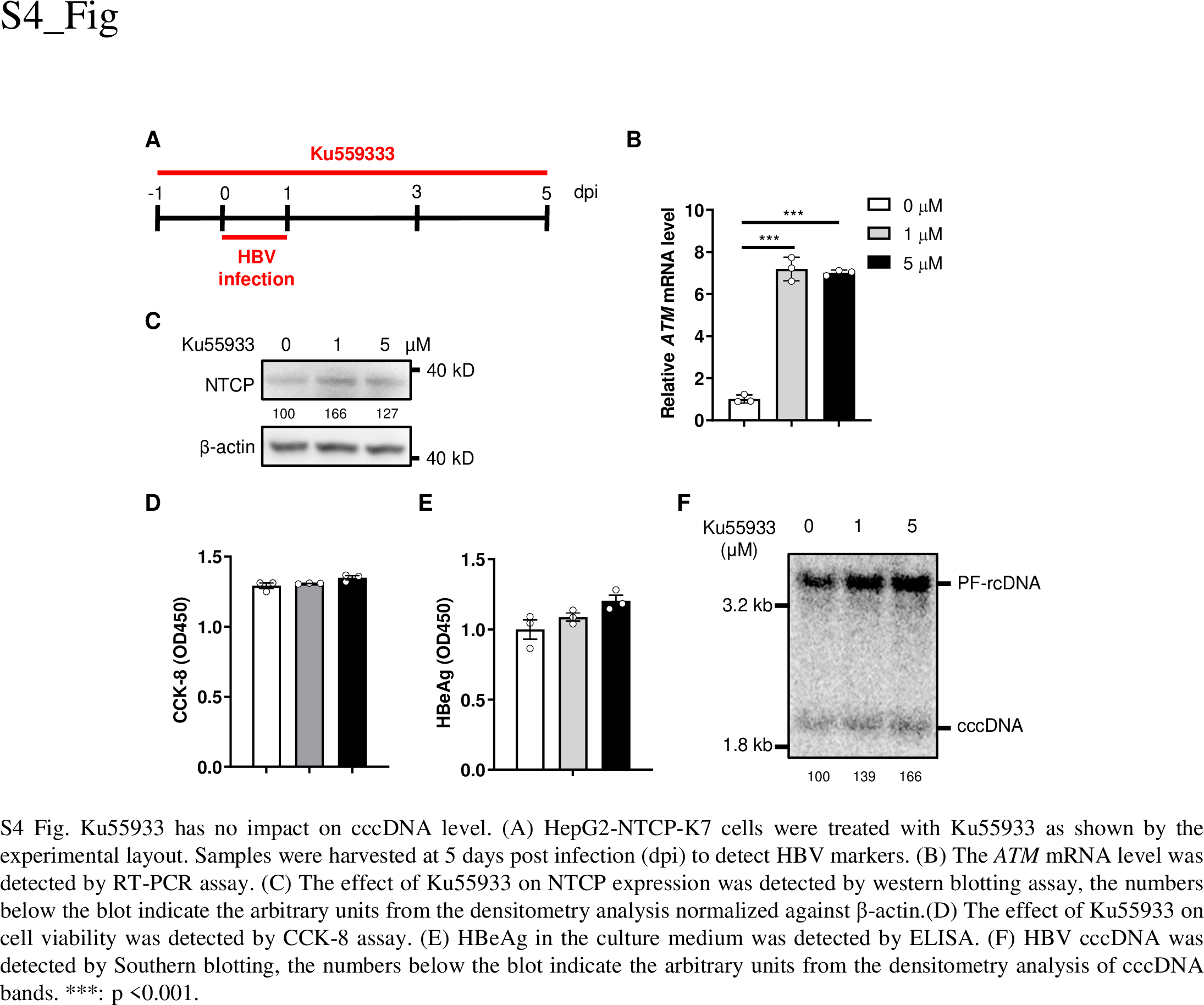

Supplement: S4 Fig — (A) HepG2-NTCP-K7 cells were treated with Ku55933 as shown by the experimental layout. Samples were harvested at 5 days post infection (dpi) to detect HBV markers. (B) The ATM mRNA level was detected by RT-PCR assay. (C) The effect of Ku55933 on NTCP expression was detected by western blotting assay, the numbers below the blot indicate the arbitrary units from the densitometry analysis normalized against β-actin.(D) The effect of Ku55933 on cell viability was detected by CCK-8 assay. (E) HBeAg in the culture medium was detected by ELISA. (F) HBV cccDNA was detected by Southern blotting, the numbers below the blot indicate the arbitrary units from the densitometry analysis of cccDNA bands. ***: p <0.001. (TIF) [file ppat.1012824.s004.tif]

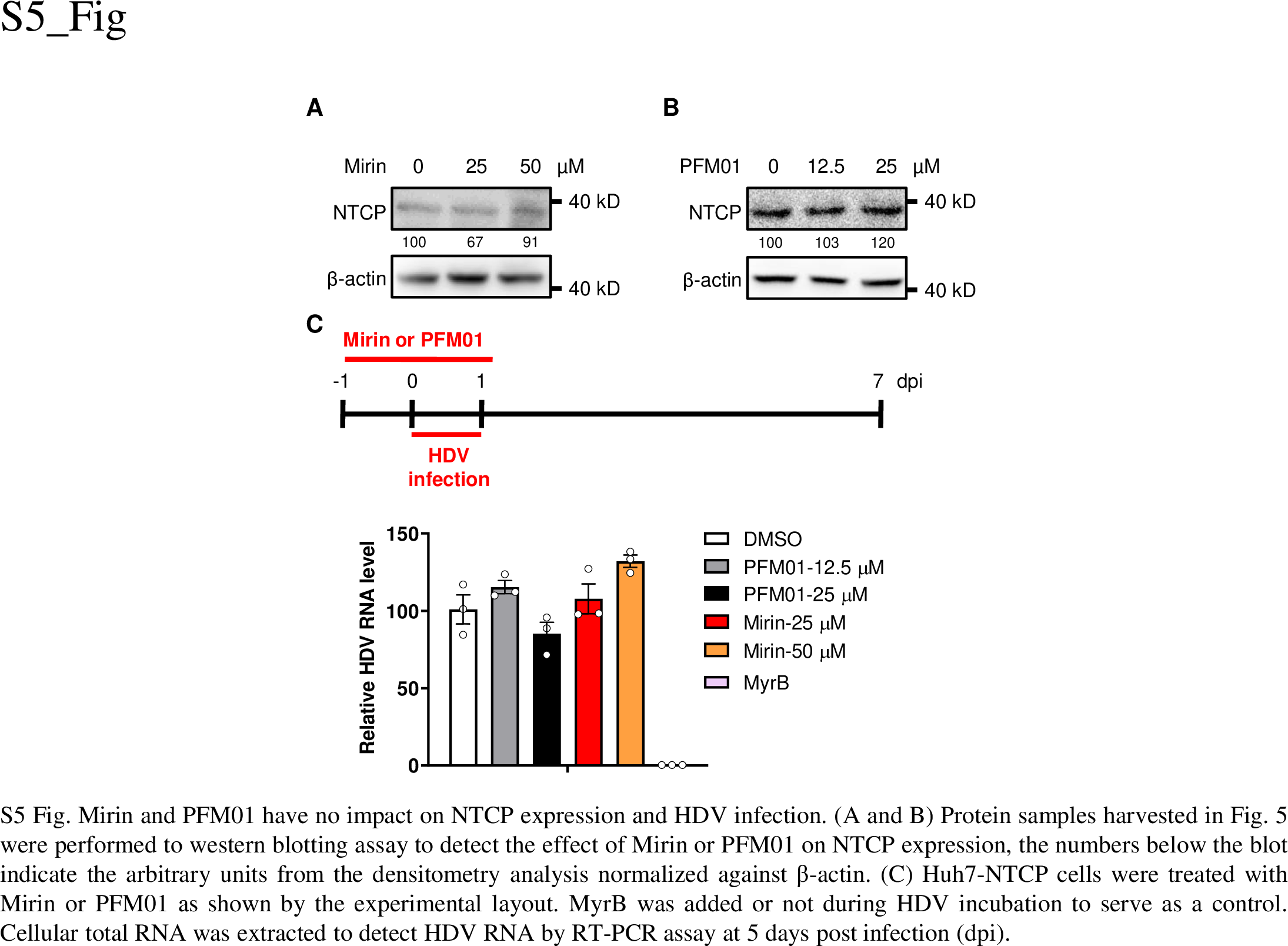

Supplement: S5 Fig — (A and B) Protein samples harvested in Fig 5 were performed to western blotting assay to detect the effect of Mirin or PFM01 on NTCP expression, the numbers below the blot indicate the arbitrary units from the densitometry analysis normalized against β-actin. (C) Huh7-NTCP cells were treated with Mirin or PFM01 as shown by the experimental layout. MyrB was added or not during HDV incubation to serve as a control. Cellular total RNA was extracted to detect HDV RNA by RT-PCR assay at 5 days post infection (dpi). (TIF) [file ppat.1012824.s005.tif]

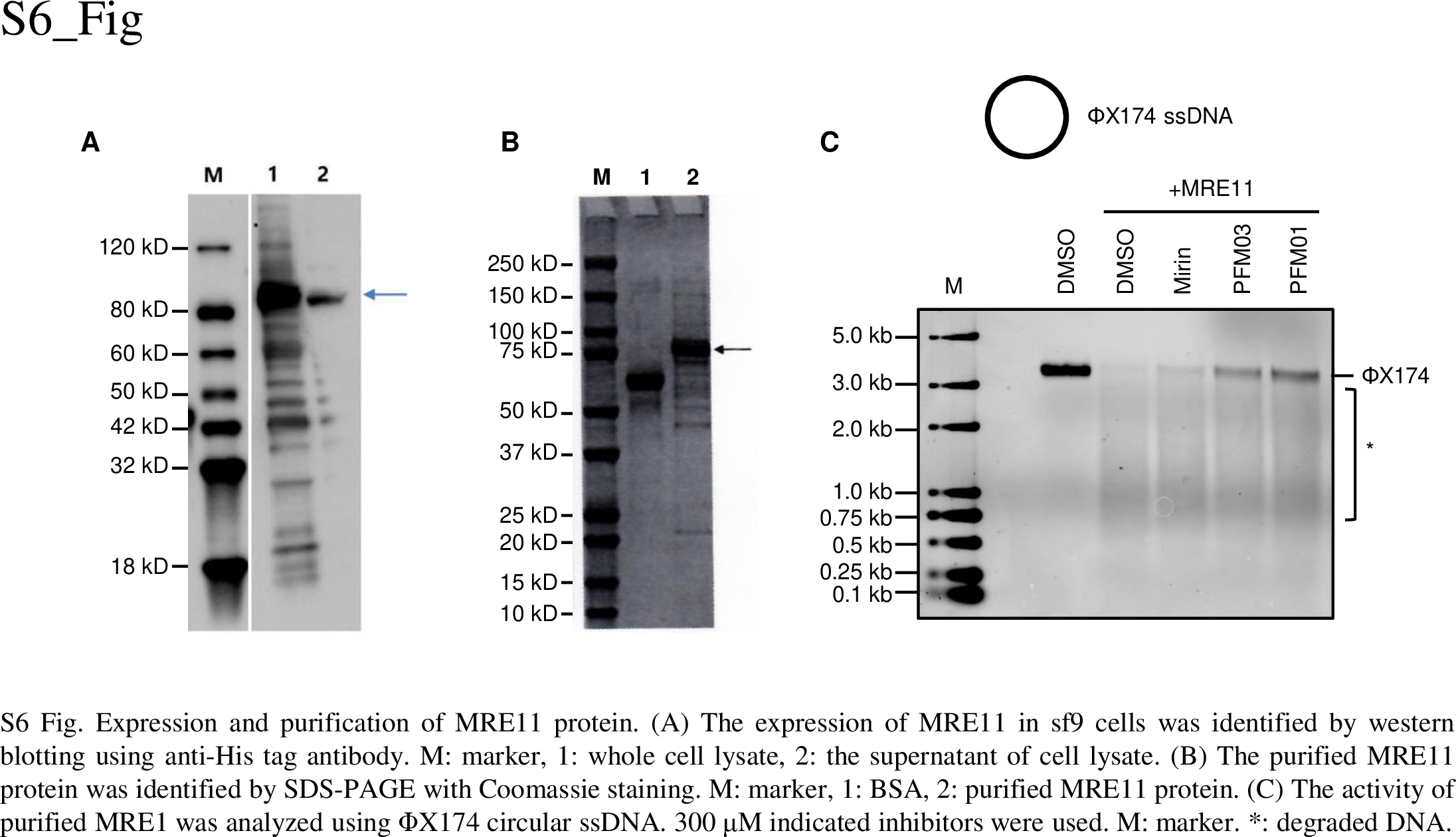

Supplement: S6 Fig — (A) The expression of MRE11 in sf9 cells was identified by western blotting using anti-His tag antibody. M: marker, 1: whole cell lysate, 2: the supernatant of cell lysate. (B) The purified MRE11 protein was identified by SDS-PAGE with Coomassie staining. M: marker, 1: BSA, 2: purified MRE11 protein. (C) The activity of purified MRE1 was analyzed using ΦX174 circular ssDNA. 300 μM indicated inhibitors were used. M: marker. *: degraded DNA. (TIF) [file ppat.1012824.s006.tif]

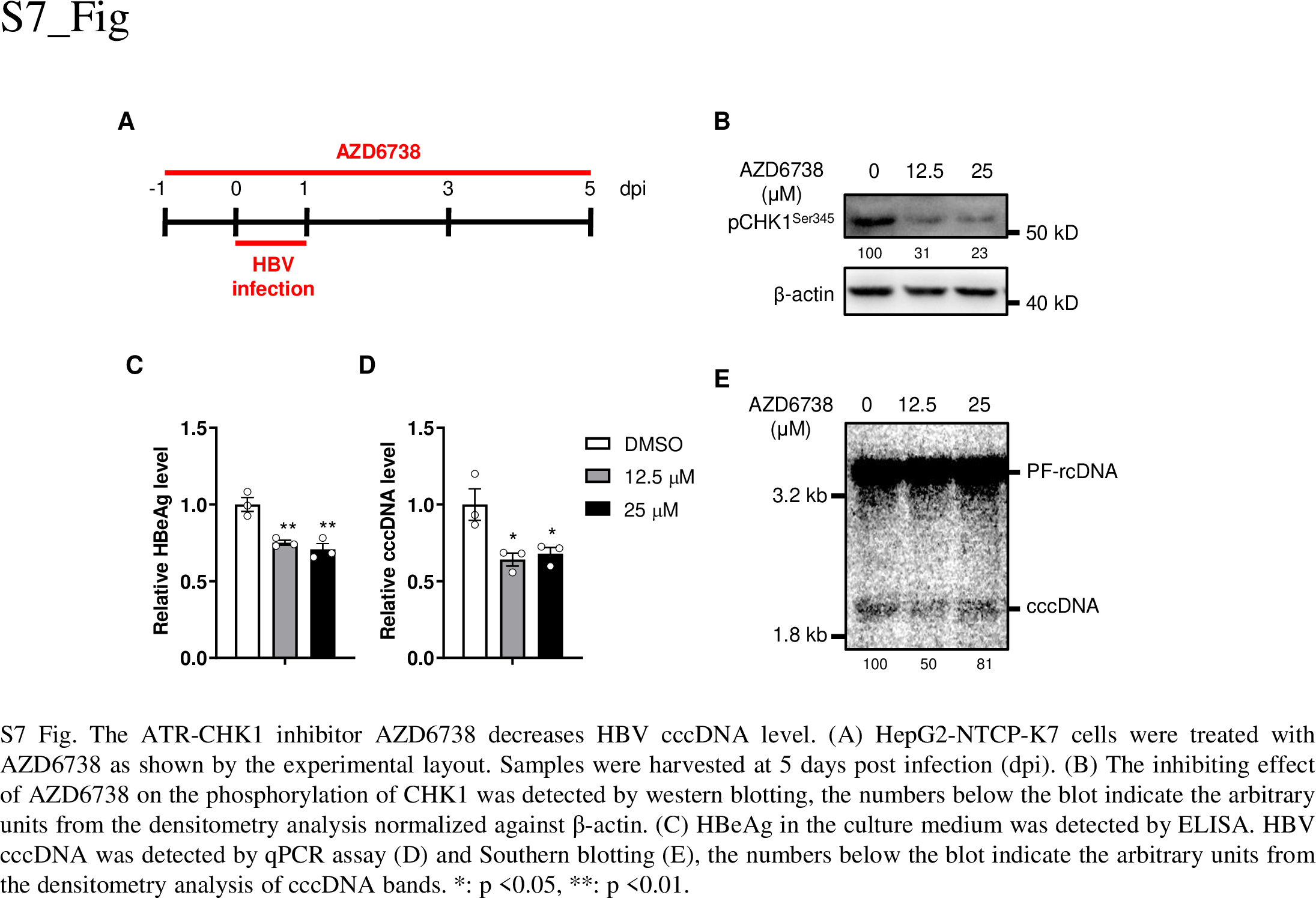

Supplement: S7 Fig — (A) HepG2-NTCP-K7 cells were treated with AZD6738 as shown by the experimental layout. Samples were harvested at 5 days post infection (dpi). (B) The inhibiting effect of AZD6738 on the phosphorylation of CHK1 was detected by western blotting, the numbers below the blot indicate the arbitrary units from the densitometry analysis normalized against β-actin. (C) HBeAg in the culture medium was detected by ELISA. HBV cccDNA was detected by qPCR assay (D) and Southern blotting (E), the numbers below the blot indicate the arbitrary units from the densitometry analysis of cccDNA bands. *: p <0.05, **: p <0.01. (TIF) [file ppat.1012824.s007.tif]
