# Supplementary material for: Hepatitis B virus hijacks MRE11–RAD50–NBS1 complex to form its minichromosome
Source: PLoS Pathog. 2025 Jan 3;21(1):e1012824. doi: 10.1371/journal.ppat.1012824 (PMC11734937; doi:10.1371/journal.ppat.1012824)
Supplement: Attachment — Submitted filename: point-by-point response-1116-final.docx [file ppat.1012824.s009.docx]

We thank the reviewers and editor for their helpful comments and are pleased that the reviewers regarded our work as “well conceived and executed”, “convincing”, “well written and easy to follow”. In this version, we have performed additional experiments including RAD50 mutant overexpression, MRE11 nuclease assays, in vitro cccDNA synthesis assay with RAD50 depletion, intracellular cccDNA amplification assay and addressed all the reviewers’ concerns in a proper way. We believe that our paper has improved considerably. Please find out point-by-point response below in *italic blue*.

**Part I – Summary**

Please use this section to discuss strengths/weaknesses of study, novelty/significance, general execution and scholarship.

Reviewer #1: The authors of this paper study the role of MRE11-RAD50-NBS1 complex on HBV infection. Zhao, Wang et al. demonstrated that si/shRNA-mediated knock-down of this complex reduces HBV infection parameters in HepG2-NTCP and primary human hepatocytes. Furthermore, the authors show that chemical compounds that inhibit different activities of the complex (Mirin and PFM1) decreased HBV infection without having any impact in cell viability. The text is well written and easy to follow.

Reviewer #2: In this manuscript, Zhao and colleagues provide several lines of evidence supporting the notion that MRN complex participates in the formation of HBV cccDNA via activation of ATR-CHK1 pathway during HBV infection. While the role of MRN complex in cccDNA formation is strongly supported by genetic and pharmacological evidence, the molecular mechanism underlying the interaction of MRN and ATR-CHK1 pathway in cccDNA synthesis remains to be determined.

Overall, the study is well conceived and executed. The conclusions are generally supported by the data presented.

Reviewer #3: The persistence of HBV covalently closed circular DNA (cccDNA) is the basis of chronic HBV infection and the major obstacle to an HBV cure. cccDNA is formed through repairing the HBV partially double-stranded, relaxed circular DNA (rcDNA), derived from infecting virions (during de novo infection) or newly made cytoplasmic mature nucleocapsids (during intracellular cccDNA amplification). To study the requirements of cccDNA formation from rcDNA, the authors took advantage of an in vitro rcDNA repair system whereby they isolated host factors interacting with rcDNA and identified the host proteins by mass spectrometry. They found that the host cell MRE11–RAD50–NBS1 (MRN) complex as one such potential factor. Transient or stable knockdown of MRE11, RAD50 or NBS1 in cells before HBV infection significantly decreased viral markers, including cccDNA, while restoration of these proteins reversed the effects. Chromatin immunoprecipitation assay validated the interaction of MRN complex and HBV DNA. On the other hand, MRN knockdown after HBV infection showed no effect on viral replication, suggesting that the MRN complex inhibited the formation of cccDNA without affecting its stability or transcriptional activity. Mirin, a MRN complex inhibitor which blocks the exonuclease activity of MRE11 and MRN-dependent activation of ATM, but not an ATM kinase inhibitor KU55933, could decrease cccDNA level. Likewise, the MRE11 endonuclease activity inhibitor PFM01 treatment decreased cccDNA. Furthermore, the inhibition of ATR-CHK1 pathway, which is known to be involved in cccDNA formation, impaired the effect of MRN complex on cccDNA. Similarly, inhibition of MRE11 endonuclease activity mitigated the effect of ATR-CHK1 pathway on cccDNA. These findings indicate that the MRN complex cooperates with ATR-CHK1 pathway to regulate the formation of HBV cccDNA.

The authors’ results thus appear to indicate that the host cell MRN complex is required for cccDNA formation during HBV infection. The data are largely consistent and convincing. However, there are a number of issues that needs to be resolved.

**Part II – Major Issues: Key Experiments Required for Acceptance**

Please use this section to detail the key new experiments or modifications of existing experiments that should be absolutely required to validate study conclusions.

Generally, there should be no more than 3 such required experiments or major modifications for a "Major Revision" recommendation. If more than 3 experiments are necessary to validate the study conclusions, then you are encouraged to recommend "Reject".

Reviewer #1: • Line 75 POLδ should be included with the rest of the polymerases as shown in citation 12

*Thanks for your suggestion. We have added this information (Line 75).*

• Overall, we will recommend quantifying both WB and Southern blots in all the experiments.

*Thanks for your suggestion. We have quantified the bands of both WB and Southern blots and presented the values in all images.*

• Figure 1 Panel E: In the chromatin immunoprecipitation assay, it is not very clear that when pulling down with HBc antibody, RAD50 is also pulled down. The WB band looks different compared with the band revealed when pulling with antiRAD50 antibody.

*As the reviewer pointed out, we did not detect RAD50 or NBS1 in the pull-down using the HBc antibody. This is likely because, in infected cells, only a small fraction of the MRN complex binds to cccDNA. Thus, when enriching cccDNA with the HBc antibody, the amount of MRN complex present is too low to be easily detected. Similarly, when enriching cccDNA with the RAD50 antibody, we also did not detect HBc. This is likely because the majority of HBc in HBV-infected cells assembles into capsids, leaving only a small portion available to bind with cccDNA.*

• Figure 2. Panels G and M. The bands detected in the Southern blot do not have enough intensity. Please quantify the cccDNA band and express the data relative to the siNC or shNC condition.

*Thanks for your suggestion. We have quantified band intensity as suggested. In addition, a new Fig. 2M as below has been added.*

• Figure 3. To rigorously demonstrate that RAD50 overexpression rescues HBV infection in RAD50 silenced cells, we will encourage to overexpress a RAD50 mutant (e.g. RAD50 that disrupts MRN complex formation PMID: 26951044) as control or the wild type RAD50 overexpression.

*Thank you for your suggestion. We have constructed a lentiviral vector expressing the RAD50 mutant as previously described (PMID: 26951044). As shown in the results below, expression of this RAD50 mutant in RAD50 knockdown cells does not restore the levels of MRE11 and NBS1 or the levels of HBV markers. These findings have been included in the updated Fig. 3.*

• Figure 4. The downregulation of RAD50 by siRNAs shown in the WB of panel B is less efficient compared with previous WB. Therefore, it is possible that RAD50 silencing didn’t have an impact in HBV infected cells because the efficiency of silencing was not enough. We will encourage the authors to perform this experiment in shNC and shRAD50-1 silenced cells.

*Thank you for your suggestion. We used lentiviruses expressing either shNC or shRAD50 (Lenti-shNC and Lenti-shRAD50-1) to knock down RAD50 expression. As shown in the new Fig.4, RAD50 expression was significantly reduced; however, HBV infection remained unaffected. These findings suggest that RAD50 does not influence cccDNA transcription or stability.*

• Figure 8. We will encourage the authors to eliminate this figure as may confuse the reader.

*Thank you for your suggestion. We have removed Figure 8.*

• The authors do not include enough data supporting the mechanism by which the MRN complex repairs HBV rcDNA in HBV infection. There is no data supporting that cccDNA biogenesis occurs through 5’ end resection of minus strand forming truncated rcDNA.

*Our results in new Figure 8 showed that MRN complex can cooperate ATR-CHK1 pathway to participate in regulating the formation of HBV cccDNA. Inhibition of ATR-CHK1 pathway induced accumulation of novel processing product from RC DNA-5’ truncated minus strands (PMID:32071277). Hence, the RC DNA-5’ truncated minus strands should be detected. we tried to detect this truncated rcDNA using terminal RACE assay and Southern blotting. Unfortunately, perhaps due to some technical problems, we did not detect this rcDNA. In addition, to further uncover the mechanism by which the MRN complex repairs HBV rcDNA, we used the purified MRE11 to perform the nuclease assays in vitro. The results showed that MRE11 can directly digest different rcDNA intermediates through its endo- and exo-nuclease activities, indicating that HBV rcDNA is a substrate of MRE11.*

• Supplementary figure 3. The knockdown efficiency of different siRNAs and the impact on HBV infection does not always correlate.

*We believe two possible reasons may explain this outcome. First, the MRN complex has multiple functions. It plays a central role in the initiation and maintenance of cellular responses to DNA double-strand breaks, stalled replication forks, dysfunctional telomeres, and viral DNA infections (PMID: 29709199). Knockdown of DNA repair factors can have pleiotropic effects on cells, potentially impacting the repair of rcDNA indirectly, such as by altering the cell cycle (PMID: 34452329). The second possibility is that the siRNAs may affect the expression of unknown genes. To investigate this, we used the NCBI database to align the siRNA sequences. The results, shown below, confirm that all the siRNAs target the correct genes. However, it remains possible that these siRNAs could influence the expression of other genes through potential off targeting.*

*siMRE11-1: TGTTGGTTTGCTGCGTATTAA*

*siMRE11-2: ACGACTGCGAGTGGACTATAG*

*siMRE11-3: GTTGAGGGAAAGAGCTTATAA*

*siNBS1-1: CCATCCCAGTACAGGATTAAA*

*siNBS1-2: GCAAGCAGATACATGGGATTT*

*siNBS1-3: GCTTATTTAGAGTCCTAGTTT*

Reviewer #2: 1. It will be interesting to test whether depletion of MRN components from the cell lysates impairs cccDNA formation in the in vitro cccDNA synthesis assay.
*Thank you for your suggestion. We depleted the MRN components from the cell lysates using a RAD50 antibody, as described previously (PMID: 32152586). The results show that RAD50 and NBS1 were nearly depleted from the cell extracts. However, the repair of rc precursor (rcDNA-II), RrcDNA (rcDNA-I), and NA-rcDNA remained unaffected. In addition, we also prepared cell extracts using HepG2-NTCP-shNC and HepG2-NTCP-shPOLK cell lines to examine the roles of POLK on cccDNA formation in this vitro system. Unfortunately, the results of two repetitions showed that knockdown of POLK did not affect the cccDNA formation in this vitro assay, which is not consistent with the results in the infection system (PMID: 27783675). One possibility is that the small amount of residual MRN in the human cytoplasmic extracts is sufficient to mediate cccDNA formation. Another explanation is that this cell lysate repair system may not fully represent the cccDNA formation process in infected cell nuclei, where the expression levels, subcellular localization, and accessibility of certain host factors are likely to be more complex in a true infection system.*

2. Why are the HBV DNA species, particularly PF rcDNA and dslDNA, on Hirt DNA Southern blots shown in Figs. 2, 3 and 4, especially 4H, very different?

*The differences in HBV DNA species, particularly PF-rcDNA and dslDNA, observed in the Hirt DNA Southern blots (Figs. 2, 3, and especially 4H) could be attributed to variations in extraction and testing batches. Unlike cccDNA, PF-rcDNA and dslDNA are not stable structures and are more prone to degradation during extraction and electrophoretic separation. We have repeated some of these experiments, and the updated results are now presented in the new Fig. 2M and Fig.4G shown below.*

*Fig. 2M	Fig. 4G*

3. It is not clear how MRN complex, particularly the MER11 exo- and/or endo-nuclease activity connect to ATR-CHK1 pathway activation and cccDNA synthesis. Further dissection of relationship between MRN complex and ATR-CHK1 pathway shall reveal important molecular insight on cccDNA synthesis.

*Thanks for your suggestion. As we responded earlier, our results in Figure 8 showed that MRN complex can cooperate ATR-CHK1 pathway to participate in regulating the formation of HBV cccDNA. Inhibition of ATR-CHK1 pathway induced accumulation of novel processing product from RC DNA-5’ truncated minus strands (PMID:32071277). Hence, the 5’-truncated minus strands of RC DNA should be detectable. We attempted to identify this truncated rcDNA using a terminal RACE assay and Southern blotting. However, due to potential technical issues, we were unable to detect this rcDNA. Additionally, to further explore the mechanism by which the MRN complex repairs HBV rcDNA, we performed in vitro nuclease assays using purified MRE11. The results showed that MRE11 can directly digest various rcDNA intermediates through its endo- and exonuclease activities, indicating that HBV rcDNA is a substrate of MRE11.*

Reviewer #3: 1. What are the effects of the MRN complex on intracellular cccDNA amplification? Given that previous reports suggest that different host factors may be involved in cccDNA formation during de novo infection vs. amplification, it would be important to compare the effects of MRN on these two alternative pathways of cccDNA formation.

*Thank you for your suggestion. Following a previous study (PMID: 31026293), we knocked down RAD50 expression or inhibited MRE11 nuclease activity using Mirin and PFM01 to assess intracellular cccDNA amplification in Huh7 cells transfected with the HBV replication-competent plasmid pCMV-HBV-ΔL. As a positive control, we used AZD6738, an ATR-CHK1 pathway inhibitor known to reduce intracellular cccDNA amplification (PMID: 32071277). The results showed that neither knocking down RAD50 nor inhibiting MRE11 nuclease activity affected intracellular cccDNA amplification.*

*(Above: core-associated DNA. Below: Hirt DNA. Hirt DNA was firstly digested with DpnI to remove plasmids, then was treated with 85℃ for5 min to denature PF-rcDNA to ssDNA, EcoRI digestion was performed at last to linearize cccDNA)*

2. What are the effects of depleting MRN in the cell-free system on cccDNA formation? This seems to be a missed opportunity that the authors should have obviously taken advantage of.

*Thanks for the suggestion. Please see our response to Reviewer 2 Ponit 1.*

3. Fig. S1. This figure needs the essential time zero control.

*Thanks for your suggestion. We repeated the experiment with time zero control. The data is updated in the revised manuscript.*

**Part III – Minor Issues: Editorial and Data Presentation Modifications**
Please use this section for editorial suggestions as well as relatively minor modifications of existing data that would enhance clarity.

Reviewer #1: Typo in line 389. “In the presence of of” : should be “in the presence of”

*Thanks for your suggestion. We have corrected it. (Line 421)*

Reviewer #2: 1. Line 271, should be top-listed “proteins”. Similarly, the “genes” should be replaced with “proteins” in the following two sentences.
*Thanks for your suggestion. We have corrected it. (Lines 285-288)*

2. The discussion on MER11 inhibitors for cancer therapy can be significantly shorten or removed.

*Thanks for your suggestion. We have removed the discussion.*

Reviewer #3: 1. Fig. 2M. The Southern blot image is of poor quality and needs to be replaced with a better image blot.

*Thanks for your suggestion. We have added a new figure as shown below.*

2. Fig. S3. Why was cccDNA reduction not correlated with NBS1/1-3 siRNA knockdown effect?

*Thanks for pointing this issue out. As we reply to the same issue raised by Reviewer 1, we believe two possible factors may explain this outcome. First, knockdown of DNA repair factors can have pleiotropic effects on cells, potentially impacting rcDNA repair indirectly, such as through alterations in the cell cycle. The second possibility is that the siRNAs may off target the expression of unknown genes.*
