# Supplementary material for: Hepatitis B virus hijacks MRE11–RAD50–NBS1 complex to form its minichromosome
Source: PLoS Pathog. 2025 Jan 3;21(1):e1012824. doi: 10.1371/journal.ppat.1012824 (PMC11734937; doi:10.1371/journal.ppat.1012824)
Supplement: Attachment — Submitted filename: point-by-point response-1212-xyc.docx [file ppat.1012824.s010.docx]

Please find out point-by-point response below in *italic blu*e.

**Reviewers' Comments:**
Reviewer's Responses to Questions

**Part I - Summary**
Please use this section to discuss strengths/weaknesses of study, novelty/significance, general execution and scholarship.

Reviewer #1: The authors have generally addressed my comments/concerns from the prior round of review. I just have one point on the new figure 8A/B: Showing that purified MRE11 degrades rcDNA does not fit well with the data shown before. The authors need to explain better how rcDNA can be a substrate for the MRN complex resulting in either cccDNA biogenesis or rcDNA degradation.

*Thanks for pointing this issue out. Our new findings demonstrate that rcDNA can serve as a substrate for MRE11 and be degraded by purified MRE11.* *While this degradation does not appear to promote cccDNA formation,* *the nuclease activity of MRE11 is regulated within the context of the MRN complex. For instance, NBS1 has been shown to inhibit MRE11/RAD50-catalyzed 3′–5′ exonucleolytic degradation of clean DNA ends, thereby preventing excessive 3′ strand removal (PMID: 27814491). Furthermore, replication protein A (RPA), which was enriched in the rcDNA pull-down assay (Fig. 1C), is known to strongly inhibit EXO1 activity by binding to the resulting single-stranded DNA (PMID: 26884156, PMID: 14636568). To provide further clarity, we have expanded the discussion section of the manuscript (Lines 517–538) and proposed a potential mechanism by which the MRN complex may contribute to rcDNA repair.*

Reviewer #2: The authors have addressed my comments on the manuscript with satisfaction.

Reviewer #3: The revised manuscript addressed all my concerns.

**Part II – Major Issues: Key Experiments Required for Acceptance**
Please use this section to detail the key new experiments or modifications of existing experiments that should be absolutely required to validate study conclusions.

Generally, there should be no more than 3 such required experiments or major modifications for a "Major Revision" recommendation. If more than 3 experiments are necessary to validate the study conclusions, then you are encouraged to recommend "Reject".

Reviewer #1: (No Response)

Reviewer #2: No.

Reviewer #3: (No Response)

**Part III – Minor Issues: Editorial and Data Presentation Modifications**
Please use this section for editorial suggestions as well as relatively minor modifications of existing data that would enhance clarity.

Reviewer #1: (No Response)

Reviewer #2: No.

Reviewer #3: (No Response)
